# Production of the Polyhydroxyalkanoate PHBV from Ricotta Cheese Exhausted Whey by *Haloferax mediterranei* Fermentation

**DOI:** 10.3390/foods9101459

**Published:** 2020-10-14

**Authors:** Susanna Raho, Vito Emanuele Carofiglio, Marco Montemurro, Valerio Miceli, Domenico Centrone, Paolo Stufano, Monica Schioppa, Erica Pontonio, Carlo Giuseppe Rizzello

**Affiliations:** 1 EggPlant S.r.l., 70044 Polignano a Mare, Italy; susanna.raho@gmail.com (S.R.); ve.carofiglio@gmail.com (V.E.C.); domenicocentrone@gmail.com (D.C.); paolo@eggplant.it (P.S.); 2 Department of Soil, Plant and Food Science, University of Bari Aldo Moro, 70125 Bari, Italy; marco.montemurro@uniba.it (M.M.); erica.pontonio@uniba.it (E.P.); 3 ENEA Research Centre, Department for Sustainability, 72100 Brindisi, Italy; valerio.miceli@enea.it (V.M.); monica.schioppa@enea.it (M.S.)

**Keywords:** whey, ricotta cheese exhausted whey, *Haloferax*, bioplastic, PHA

## Abstract

In the last decade, the dairy industry underwent a rapid expansion due to the increasing demand of milk-based products, resulting in high quantity of wastewater, i.e., whey and ricotta cheese exhausted whey (RCEW). Although containing high content of nutritional compounds, dairy by-products are still disposed as waste rather being reintroduced in a new production chain, hence leading to environmental and economic issues. This study proposes a new biotechnological approach based on the combination of membrane filtration and fermentation to produce poly-hydroxyalkanoates (PHA), biodegradable bioplastics candidate as an alternative to petroleum-derived plastics. The protocol, exploiting the metabolic capability *Haloferax mediterranei* to synthesize PHA from RCEW carbon sources, was set up under laboratory and pilot scale conditions. A multi-step fractionation was used to recover a RCEW fraction containing 12.6% (*w*/*v*) of lactose, then subjected to an enzymatic treatment aimed at releasing glucose and galactose. Fermentation conditions (culture medium for the microorganism propagation, inoculum size, time, and temperature of incubation) were selected according to the maximization of polymer synthesis, under in-flasks experiments. The PHA production was then tested using a bioreactor system, under stable and monitored pH, temperature, and stirring conditions. The amount of the polymer recovered corresponded to 1.18 g/L. The differential scanning calorimetry (DSC) analysis revealed the poly(3-hydroxybutyrate-co-3-hydroxyvalerate) (PHBV) as the polymer synthesized, with a relatively high presence of hydroxyvalerate (HV). Identity and purity of the polymer were confirmed by attenuated total reflectance-Fourier transform infrared (ATR-FTIR) and X-ray photoelectron (XPS) spectroscopy analyses. By combining the fractionation of RCEW, one of the most abundant by-products from the agri-food industry, and the use of the halophile *Hfx mediterranei*, the production of PHBV with high purity and low crystallinity has successfully been optimized. The process, tested up to pilot scale conditions, may be further implemented (e.g., through fed-batch systems) and used for large-scale production of bioplastics, reducing the economical and environmental issues related the RCEW disposal.

## 1. Introduction

In the last decade, the dairy industry underwent a rapid expansion due to the increasing demand of milk-based products, resulting in high quantity of wastewater and by-products [1,2,3,4,5]. Consequently, the sector is facing the waste disposal problem, mainly determined by the related high pollutant load.

Dairy waste treatments include mechanical, physicochemical, and biological methods [4]. Overall, they are complex, expensive, and time-consuming, and the reason why waste is often disposed illegally in the environment, even if the industries must follow stringent national and community regulations [6]. A zero-waste approach is urgently required to overcome this issue.

The main dairy derived by-products are cheese whey, ricotta cheese exhausted whey (RCEW), and buttermilk. Whey is a cheese manufacturing-based by-product, derived by precipitation and separation of milk casein and lipid from whole milk. It represents 85–95% of the total processed milk’s volume [7], resulting in 9 L of whey for each kg of cheese produced [1]. About 200 million tons of whey per year are globally produced [8], of which 7–9.5 × 10^6^ tons come only from Italy [9].

Part of the whey is commonly used to produce ricotta cheese, with an alternative eco-friendly (and traditional) food technology. Whey proteins undergo the thermal coagulation (80–90 °C), a very low-efficiency process (about 2–3%) from which however results a large amount of RCEW. This by-product is extensively produced in South Europe, of which 1 million tons for year only in Italy [10].

RCEW composition is similar to whey, but it is depleted in fats and proteins and enriched in salts, organic acids, and lactose [11]. Due to the high organic content (4% *w*/*v* of lactose) [12], the RCEW is extremely polluting and is characterized by considerable chemical (COD) and biochemical (BOD) oxygen demand values, of about 40 g/L and 66 g/L, respectively [13].

The high sugar content potentially allows the use of RCEW as a suitable matrix for the biotechnological production of biodiesel [14], bioethanol [15], probiotics [16], lactic acid [17], lactobionic acid, growth medium for lipid production [14], and fermented drink [18].

Although the conversion of whey and RCEW in food, feed, and pharmaceutical and cosmetic ingredients has largely been investigated [19], more than 50% is still treated as a common waste, generating several environmental and economic problems.

Recently, the implementation of the RCEW processing through the application of filtering membrane technologies that contemporarily allow the recovery of side compounds (e.g., whey proteins and lactose) and the reduction of organic pollutant load, has been proposed [20,21,22]. To date, RCEW fractionation represents one of the most promising and innovative methods to enhance this still under-studied by-product [23,24].

In this work, a new approach to valorize RCEW was proposed. The study describes the set-up and optimization of an integrate biotechnological process based on the combination of RCEW pre-treatment (membrane process) and fermentation (with a halophile microorganism), aiming at producing poly-hydroxyalkanoates (PHA), thermo-polymers considered as an intriguing biodegradable alternative to petroleum-derived plastics [25,26].

Despite of its enormous uses and economic benefits, plastics account for significant waste accumulation, which mostly remains undegraded and recalcitrant for decades in the global environment [27]. PHA are linear polyesters synthesized by a variety of microorganisms, serving as an intracellular reservoir for energy and carbon supply [28,29]. Owing to their combined properties of biodegradability, biocompatibility, and thermoplasticity, PHA were identified as promising candidates for bio-based plastics production [30,31]. Although it has tremendous market potential, the high production costs still limit the PHA large-scale application [32].

Recently, halophiles, a diversified group of microorganisms having the ability to survive in hypersaline environments, are attracting the interest of the scientific and industrial research as a cost-effective tool to produce PHA [31,33]. The adaptation to extreme conditions confers to halophiles a great potential for PHA production. The foremost advantage is that the high salinity requirements reduces the chances of microbial contamination to a great extent [34], allowing to perform bioprocesses without expensive sterilization pre-treatments of the substrates. Moreover, microbial cells can be easily lysed in normal water due to the high intracellular osmotic pressure; thus, further reducing PHA recovery cost [35]. However, the use of halophiles for large-scale PHA production is still associated to several issues [36,37]. Among these, the treatment of the saline fermentation effluent and the corrosion of the conventional fermentation equipment due to the high salt concentration of the substrates represent the main difficulties.

Aiming at (i) solving the disposal of a highly polluting agri-food waste and (ii) finding an economic alternative way for the production of bioplastic, a biotechnological process for PHA production, based on multi-step membrane fractionation of RCEW followed by fermentation by *Haloferax mediterranei*, was here set-up and optimized.

## 2. Materials and Methods

### 2.1. Microorganism and Growth Conditions

*Haloferax mediterranei* DSM1411 was purchased from Deutsche Sammlung von Mikroorganismen und Zellkulturen culture collection (DSMZ, Braunschweig, Germany) [38,39]. The microorganism was cultivated in synthetic highly saline media Halobacterium Medium 97 (HM97) and Halobacterium Medium 372 (HM372) (detailed composition, as provided by the DSMZ, is reported in Table S1) The pH of the media (determined by pH-meter 507, Crison, Milan, Italy) was adjusted to pH 7.2 with ammonia solution (0.1 M). Media were sterilized at 121 °C for 15 min prior to use. The strain was daily propagated in shake flasks, at 37 °C for 24 h, under stirring conditions (orbital shaker at 150 rpm). Inoculum consisted of 20% (*v*/*v*) of the fresh culture on total working volume. When necessary, the fresh culture was supplemented with 20% (*v*/*v*) glycerol and stored at −80 °C.

### 2.2. Multi-Step Fractionaction of the RCEW

**Figure 1:**
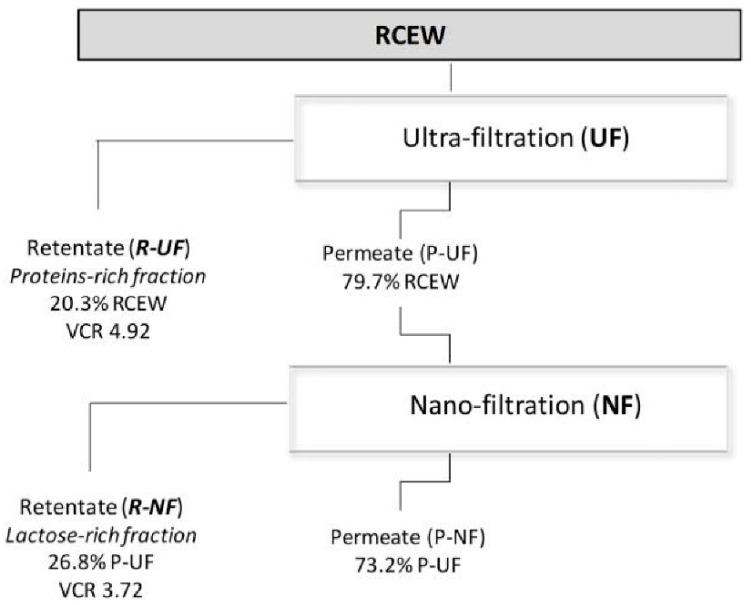
Flow-chart of fractionation process. RCEW ricotta cheese exhausted whey, R-UF and P-UF retentate and permeate after ultra-filtration (UF), R-NF and P-NF retentate and permeate after nano-filtration (NF). Fraction recover (expressed as % *v*/*v*) and Volume Concentration Ratio (VCR) were also reported.

RCEW was kindly provided by the dairy plant Caseificio dei Colli Pugliesi (Santeramo, Bari, Italy). RCEW was delivered to the fractionation pilot plant of the ENEA (Brindisi, Italy) under refrigerated conditions (4 ± 2 °C) and processed within 24 h from production [40]. The flow chart of the fractionation, based on a separative membrane process, is reported in Figure 1.

Each fractionation step led to the obtainment of aqueous permeate (P) and retentate (R), the first containing the molecules able to cross the membrane, the second containing a residual part of these and all the molecules unable to cross the membrane. The fractionation process previously set-up by Stufano et al. [41] was applied to RCEW. In details, the first step consisted in an ultra-filtration, carried out through a prototypal system equipped with a spiral wound PESH (polyethersulfone) membrane (30 kDa cut-off) ISUH030 4040 C1 (Microdyn-Nadir, Wiesbaden, Germany), spacer 44 mil (1.117 mm), area 6 m^2^. At the operating temperature (14 °C), the flow rate was 3000 L/h, with a Trans Membrane Pressure (TMP) of 3.47 bar. The permeate flow was 15.4 L/h·m^2^. The second step was a nano-filtration (NF), carried out through a prototypal system equipped with a spiral wound PESH membrane Duraslick NF 4040 (GE Healthcare, Chicago, IL, USA) with high salt rejection (MgSO_4_ 98%), spacer 30 mil (0.762 mm), area 8.5 m^2^. At the operating temperature of 40 °C, the flow rate was 3000 L/h with a TMP of 14.0 bar. Permeate flow was 27 L/h·m^2^. The VCR was calculated as the ratio between initial feed volume and retentate volume. Retentates and permeates from each filtration step were stored at −20 °C until the analyses. All the analyses were performed in triplicate.

### 2.3. RCEW, R-NF, and P-Ultra-Filtration (UF) Characterization

The pH of RCEW and derived fractions was determined by pH-meter (507-Model). Total nitrogen (TN) was determined by applying standard ISO 8968-1 Kjeldahl-based method [42], using 6.38 as conversion factor to calculate total proteins. Sugars (glucose, galactose, and lactose) were determined by HPLC analysis, using an ÄKTA purifier HPLC (GE Healthcare) equipped with a Spherisorb-5-NH2 column (4.6 × 250, Waters, Sesto San Giovanni, Italy) and a refractive index detector (RI-101, Perkin Elmer, Shelton, CT, USA), using a solution of acetonitrile/water (ratio 65:35) as mobile phase [43]. Freeze-dried sample were resuspended in 1 mL of acetonitrile (65% *v*/*v*) before analysis. The identification of the sugars and the calibration curves were obtained using commercial standards of lactose, glucose, and galactose (Sigma Aldrich, Milano, Italy). The total free amino acids (TFAA) concentration was determined using a Biochrom 30 series Amino Acid Analyzer (Biochrom Ltd., Cambridge Science Park, UK) with a Li-cation-exchange column (0.46 cm internal diameter), by post column derivatization with ninhydrin, as described by Rizzello et al. [44]. Fat and ash were determined through the international standards methods Gerber [45] and AOAC 945.46 [46], respectively. The sodium chloride content in R-NF was measured by a Sherwood 926 chloride-analyzer (Sherwood Scientific, Cambridge, UK).

BOD_5_ and COD were determined with the standard methods 5210D [47] and ISO 15705:2002 [48], respectively.

### 2.4. Set-Up of R-NF Fermentation Protocol

#### 2.4.1. Enzymatic Pre-Treatment

Due to the *Hfx. mediterranei* DSM1411 incapability to metabolize lactose, an in situ enzymatic hydrolysis of the disaccharide was assessed in R-NF. Two different liquid commercial preparations of β-galactosidase were tested: Maxilact^®^ LGI 5000 (from *Kluyveromyces lactis*, 5000 Neutral Lactase Units—NLU/g) and Maxilact A4 (from *Aspergillus oryzae*, 5000 Acid Lactase Units—ALU/g), provided by Royal DSM N.V. (Heerlen, The Netherlands). To select a proper enzyme-substrate ratio, amounts of 100, 400, and 1000 µL/100 mL of each enzyme were added to R-NF. As suggested by the enzyme provider, the mixtures were incubated at 37 °C for 5 h after adjustment of the pH to the optimal values (6.5 and 4.5 for Maxilact LGI 5000 and Maxilact A4, respectively). Residual lactose, expressed as percentage (%) of the initial concentration, before and after the incubation was determined by HPLC as described before. Water activity (aw) was determined at 25 °C with an AquaLab 4TE Dew Point water activity meter (Decagon Devices Inc., Pullman, WA, USA).

#### 2.4.2. Selection of the Fermentation Conditions

R-NF was used as substrate for the cultivation of *Hfx. mediterranei* DSM1411 without other carbon source supplementations, after β-galactosidase treatment. Micronutrients were supplemented with 1% *v*/*v* of trace element solution SL6 as described by Koller et al. [49]. The composition of SL6 solution was (g/L): ZnSO_4_ 7H_2_O, 0.1 g, MnCl_2_ 4H_2_O 0.025 g, H_3_BO_3_ 0.3 g, CoCl_2_ 6H_2_O 0.2 g, CuSO_4_ 0.006 g, NiCl_2_ 6H_2_O, 0.02 g, and Na_2_MoO_4_ H_2_O 0.03 g. The pH of R-NF was adjusted to pH 7.0–7.2 with ammonia solution. To maintain the optimal osmotic conditions for *Hfx. mediterranei* [37], the R-UF fraction was supplemented with NaCl. According to previous findings [37] the high salt concentration allowed to carry out the experiments in non-sterile conditions. Nevertheless, contaminations of other non-halophilic microorganisms were excluded by regular microscopic observations.

The set-up of the fermentation protocol included the evaluation of the *Hfx. mediterranei* DSM1411 biomass and polymer production in R-NF (details are reported below) when following parameters changed:

- medium for inoculum propagation (24 h-cultures were obtained in HM97 or HM372);
- inoculum size (10 or 20% *v*/*v*);
- NaCl supplementation (10 or 20% *w*/*v*);
- addition of yeast extract (YE) (0 or 5 g/L);
- incubation temperature (37 or 45 °C).

All experiments were carried out in 1 L flasks (containing 250 mL of substrate) and incubated in orbital shaker (150 rpm) for 72–144 h.

Aw was determined on the substrates as described above. Each condition was obtained and analyzed in three replicates.

### 2.5. Determination of Cell Dry Mass (CDM) and Poly(3-hydroxybutyrate-co-3-hydroxyvalerate) PHBV Production

At the end of the incubation, cells were recovered from the liquid substrate by centrifugation (15.000× *g* for 15 min at 4 °C). Then, cells were washed with 10% *w*/*v* NaCl solution to prevent cellular lysis and were centrifuged again to remove debris. Cell pellet was dried (60 °C for at least 24 h) and weighed. Then, PHBV was extracted from dry biomass. To achieve hypo-osmotic shock and complete cell lysis, the biomass was resuspended in deionized water (20 mL for g of CDM) until the complete solubilization. After centrifugation (12,800× *g* for 20 min at 4 °C), the supernatant was discarded and the pellet was resuspended in CHCl_3_:H_2_O solution (1:1 *v*/*v*). The organic phase containing PHBV was separated and centrifuged again (12,800× *g* for 10 min at 4 °C) to remove impurities. The pellet, corresponding to raw PHBV, was recovered and weighed. The polymer yield (%) was calculated as [PHBV (mg/L)/CDM (mg/L)] × 100.

### 2.6. Scale up Fermentation Procedure on Bioreactor

*Hfx. mediterranei* DSM1411 was cultivated in a 3 L-bioreactor (Diachrom Biotechnology, Figure S1). pH value (7.2 ± 0.01) and temperature (37 ± 0.1 °C) were automatically controlled. According to the results obtained from the in-batch trials, fermentation was carried out for 120 h. The cultivation was carried out in constant airflow rate of 1 vvm (volume of air per volume of reactor per minute) and oxygen partial pressure of about 20% of air saturation (controlled by stirring variation from 300 to 600 rpm) to guarantee aerobic condition. Foaming was inhibited by the automatic addition of Antifoam A (Sigma, Milano, Italy). CDM and PHBV were determined as mentioned above. Each experimental condition was obtained and analyzed in three replicates.

### 2.7. Polymer Characterization

For the polymer characterization, raw PHBV was solubilized in CHCl_3_ and added with 10-fold amount of cold ethanol. After a centrifugation (12,800× *g* for 10 min at 4 °C), purified PHBV was recovered and subjected to further analysis. The commercial PHBV preparation ENMAT™ Y1000P, provided by TianAn Biopolymer (Ningbo, China) was included in the analysis as reference.

#### 2.7.1. Differential Scanning Calorimetry (DSC) Analysis

DSC measurements were performed on weighted samples of approximately 10 mg using a TA Instruments DSC, Discovery apparatus. Before being analyzed, samples were subjected to conditioning at 55 °C for 24 h in a vacuum oven. The specimens were sealed into aluminum pans and subjected to two heating and cooling ramp from −25 to 200 °C and down to −25 at 10 °C/min under nitrogen atmosphere. The first heating was applied to erase thermal history of the sample. The glass transition (T_g_), crystallization (T_c_) and melting (T_m_) temperatures, melting (Δ*H_m_*) and crystallization (Δ*H_m_*) enthalpies, and crystallinity degree (*X*) were obtained from the second heating scan. The crystallinity degree (*X*) of the samples was determined by applying the following equation [50]:

$$X_{c}\;\left(\%\right)=\frac{\Delta H_{m}-\Delta H_{c}\times 100}{\Delta {H^{0}}_{m}}$$

where Δ*H_m_* (J/g) is the experimental melting enthalpy, Δ*H_c_* (J/g) is the experimental crystallization enthalpy, and Δ*H*^0^*_m_* is the calculated melting enthalpy of the PHBV considering the polymer 100% crystalline, 109 J/g [51].

#### 2.7.2. Attenuated Total Reflectance-Fourier Transform Infrared (ATR-FTIR) Analysis

Aliquots of the polymer were subjected to the ATR-FTIR analysis. Spectrum was recorded using a Spectrum Two FT-IR Spectrometer (Perkin Elmer, Shelton, CT, USA) from 400 to 4000 cm^−1^, with a resolution of 1 cm^−1^ and averaged over 32 scans.

#### 2.7.3. X-ray Photoelectron Spectroscopy (XPS) Analysis

PHBV films were obtained by dissolving samples in chloroform at 50 °C, then smeared onto a clean glass surface and evaporation in desiccator containing allochroic silica gel. The resultant films had a thickness of 50 ± 5 µm.

The XPS survey and the high-resolution measurements were used to derive the surface chemical composition of the samples using a 5000 VersaProbe spectrometer (ULVAC-PHI, Kanagawa, Japan) with monochromatic Al K_α_ radiation (1486.7 eV). The calibration of binding energy (BE) scale was performed with the C1s peak at 284.6 eV from the carbon contamination layer. The surface quantification has been carried-out by using the core level XPS narrow spectra of found elements. Spectra were collected from an area of approximately 100 µm in diameter. X-ray source operated at 100W (20 kV) in the large area slot mode (200 µm × 1350 µm). Wide Scan (WS) spectra were acquired with pass energy 117.4 eV and step size 1.0 eV (57.7 eV for the spectral regions at high resolution). Background subtraction was performed using the optimized Shirley function. Peak deconvolution and the elemental concentration values were derived from peak surface areas were performed using the PHI-MultiPak 9.6.0 and CasaXPS 2.3.17 software.

#### 2.7.4. Mechanical Analysis

PHBV samples were tested for their tensile strength (Mpa), elongation at break (%) and tensile Young’s modulus (GPa) according to the ASTM D 638-type V method [52], at crosshead speed of 5 mm/min. Analysis was performed at room temperature. An Instron Universal Testing Machine model 5584 was used. Each sample was analyzed in three replicates.

### 2.8. Statistical Analysis

All the experiments and related analysis were carried out in triplicate. Data were subjected to one-way ANOVA; pair-comparison of treatment means was achieved by Tukey’s procedure at *p* < 0.05, using the statistical software Statistica 8.0 (StatSoft Inc., Tulsa, OK, USA).

## 3. Results

### 3.1. Substrate Characterization

A multi-step technology [41], including UF and NF (Figure 1) was applied to RCEW. RCEW pH was 5.2 (Table 1), while initial BOD_5_ and COD respectively corresponded to 31.0 ± 1.2 and 83.4 ± 0.9 g/L. In the first step of the fractionation, UF allowed the separation of the proteins-rich retentate (ultra-filtration retentate, R-UF) in which almost all the nitrogen and fat of the RCEW was retained. R-UF, corresponding to 20.3% *v*/*v* of the processed RCEW, was indeed characterized by estimated proteins concentration of 1.81 ± 0.05% *w*/*v* (285 ± 8 mg/L of TN), 0.75 ± 0.04% fat *w*/*v*, and 5.2 ± 0.1% *w*/*v* lactose (Table 1). The volume concentration ratio (VCR) corresponded to 4.92. The permeate from ultra-filtration (P-UF), (Table 1) was characterized by a very low TN concentration; nevertheless, a small nitrogen aliquot mainly corresponding to organic compounds having molecular mass lower than 30 kDa (peptides and free amino acids, FAA), was observed.

Indeed, TFAA concentration corresponded to 510 mg/L. Similarly to TFAA, also ash concentration resulted significantly (*p* < 0.05) higher than RCEW as the expected consequences of the partial water removal in R-UF fraction. NF allowed the recovery of 26.8% (*v*/*v*) of a lactose-rich retentate (nano-filtration retentate, R-NF), in which lactose concentration was higher than 12.5% (VCR of 3.72). TFAA also accumulated in this fraction (Table 1). An amount of 1748 ± 15 mg/L of residual chloride was found in the R-NF. BOD_5_ and COD of the R-NF, respectively corresponded to 85.5 ± 11.0 and 100.0 ± 13.0 g O_2_/L. The nano-filtration permeate (P-NF) included water and almost all salts (COD < 50 mg O_2_/L).

### 3.2. Optimization of the Microbial Growth and Poly(3-hydroxybutyrate-co-3-hydroxyvalerate) (PHBV) Synthesis Conditions

#### 3.2.1. Lactose Hydrolysis

Aiming at hydrolyzing lactose, R-NF was subjected to a β-galactosidase treatment. Two different commercial β-galactosidases from microbial sources were used. The highest rate of lactose hydrolysis (98%) was obtained after the addition of 1000 µL of Maxilact^®^ LGI 5000 per 100 mL of R-NF (Figure S2). As the consequence of the enzymatic treatment, the activity water (a_w_) decreased from 0.992 ± 0.001 (not-treated R-NF) to 0.988 ± 0.001. Treated R-NF was used as growth substrate for *Hfx. mediterranei* DSM1411 on laboratory scale without further carbon source supplementations.

#### 3.2.2. Effect of the Substrate Used for Starter Propagation

**Figure 2:**
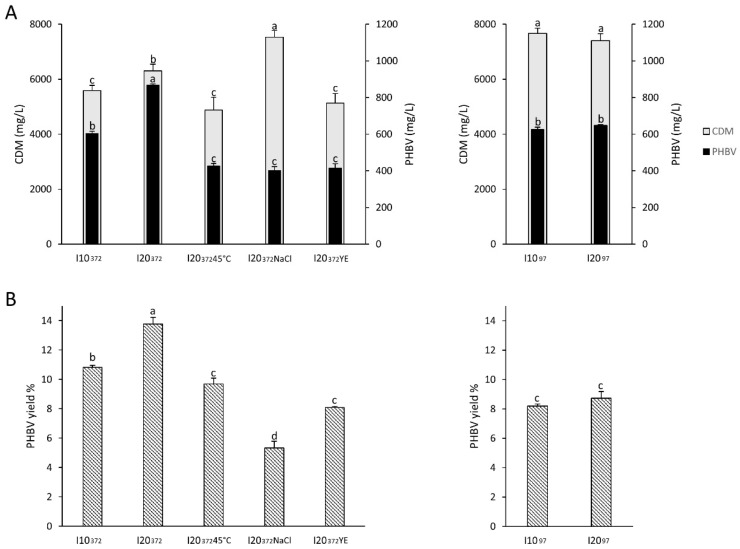
*Haloferax mediterranei* DSM1411 Cell Dry Mass (CDM) and PHBV production (panel **A**) and yield (panel **B**) in different fermentation conditions. R-NF was supplemented with 1% (*v*/*v*) SL-6 and 10% (*w*/*v*) NaCl. Inoculum was made with 10 (I10) or 20% *v*/*v* (I20) active cultures of *Haloferax mediterranei* DSM1411 grown (24 h at 37 °C) in HM372 (I10-I20_372_) or HM97 (I10-I20_97_) media. Fermentation was carried out at 37 °C for all the thesis, with the exception of I20_372_45, incubated at 45 °C. 10% *w*/*v* NaCl (final concentration 20%) or 5 g/L YE were added to the substrate inoculated with 20% of active culture in HM372 (I20_372_NaCl and I20_372_YE). In all cases, fermentation lasted 120 h. Error bars represent the standard deviation of three replicates. ^a–d^ Values in the same panel and data series, with different superscript letters, differ significantly (*p* < 0.05).

The starter was cultivated in two different media (Table S1) before inoculation, and two different amounts of the active cultures were added to the final volume of substrate to be fermented. In all the tested conditions, the fermentation medium was supplemented with 1% (*v*/*v*) SL6 and 10% (*w*/*v*) NaCl and carried out at 37 °C for 120 h. In order to evaluate the yield of PHBV, the CDM and the amount of polymer synthesized were both determined at the end of the incubation (37 °C). When HM97 was used as synthetic culture medium for the starter propagation, the highest biomass was collected (Figure 2A).

Nevertheless, the highest PHBV yield (Figure 2B) was achieved when inoculum was carried out by adding 20% (*v*/*v*) of the active culture to the substrate, and in particular when HM372 was used for the starter propagation (13.78 ± 0.5% *w*/*w*). Based on these results, the inoculum conditions including the use of HM372 and 20% (*v*/*v*) of the pre-culture as inoculum, were chosen as reference for the further comparisons (sample was coded as I20_372_).

#### 3.2.3. Effect of the Temperature

To evaluate the effect of the temperature, incubation was also carried out at 45 °C (I20_372_45). It was previously reported that 45 °C corresponds to the optimal growth temperature for *H. mediterranei*, although 37 °C allows energy saving and promotes a higher saturated oxygen concentration in the broth [53]. Compared to I20_372_, incubated at 37 °C, the temperature increase caused a significant (*p* < 0.05) decrease of the CDM (−33% *w*/*v*) and PHBV synthesis (−51% *w*/*v*) and yield (−30% *w*/*w*) (Figure 2A,B).

#### 3.2.4. Effect of Salt and Yeast Extract Supplementation

The supplementation of the substrate with additional NaCl and yeast extract (YE) was investigated. Compared to the R-NF containing only SL6 and 10% *w*/*v* NaCl (I20_372_), the aw of the substrate supplemented with NaCl (I20_372_NaCl) decreased to 0.843 ± 0.001 (vs. 0.923 ± 0.002), while no significant (*p* > 0.05) difference was found when YE was added (I20_372_YE, aw 0.923 ± 0.001). Although CDM significantly (*p* < 0.05) increased (+20%) when 20% (*w*/*v*) NaCl was used instead of 10% (Figure 2A), significant (*p* < 0.05) decrease of PHBV production (−54% *w*/*v*) and yield (−62% *w*/*w*) were observed (Figure 2B). YE supplementation led to similar results, decreases of 52% (*w*/*v*) and 39% (*w*/*w*) were respectively found for PHBV production and yield.

#### 3.2.5. Effect of the Fermentation Time

**Figure 3:**
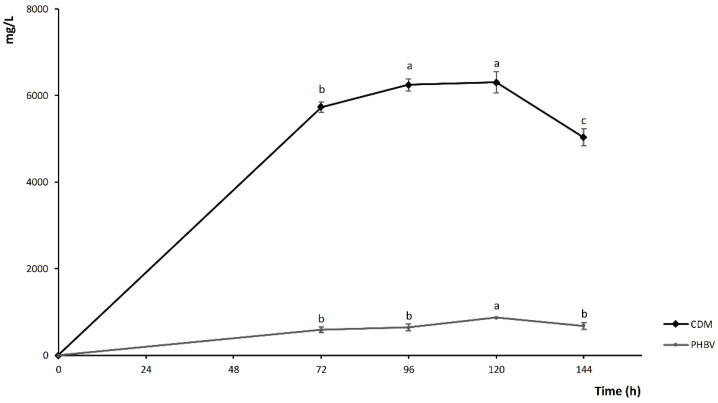
*Haloferax mediterranei* Cell Dry Mass (CDM) and PHBV production in R-NF supplemented with 1% (*v*/*v*) SL-6 and 10% (*w*/*v*) NaCl. Inoculum was made with 20% *v*/*v* active culture of *Hfx. mediterranei* DSM1411 grown (24 h at 37 °C) in HM372. Fermentation was carried out at 37 °C for 144 h. Error bars represent the standard deviation of three replicates. ^a–c^ Values in the same data series, with different superscript letters, differ significantly (*p* < 0.05).

Aiming at selecting the optimal fermentation time for polymer production, microbial growth and PHBV synthesis were monitored during a 144 h-incubation (Figure 3). No residual glucose and galactose (<0.01% *w*/*v*) were found after 72 h of incubation.

The highest CDM was obtained at 96 h of fermentation, with values stable until 120 h, while a significant (*p* < 0.05) decrease was observed at 144 h. After 72 h of incubation at 37 °C the PHBV synthesis corresponded to 591 ± 68 mg/L, while it significantly increased to 646 ± 74.21 and 868 ± 6 mg/L after 96 and 120 h of incubation, respectively (Figure 3). The PHBV yield was 10.33% (*w*/*w*) at 72 h and no significant (*p* > 0.05) differences were observed at 96 h, while the highest value was observed at 120 h, this latter corresponding to the conditions previously described for I20_372_.

After 144 h, polymer yield apparently increased up to 13.49% (*w*/*w*), due to the cell death and lysis (Figure 3), and exposure of PHBV to the action of extracellular depolymerases.

According to the results, 120 h of incubation were chosen as optimal time for the fermentation process.

### 3.3. PHBV Synthesis in Bioreactor System

Protocol for PHBV production was tested and optimized in a bioreactor system, under a continuous control of pH and temperature. All the experiments were carried out under different stirring conditions. Overall, both CDM and polymer synthesis were markedly and significantly (*p* < 0.05) higher compared to the in-flasks trials; nevertheless, the yield resulted significantly (*p* < 0.05) lower. Several authors already reported that the conditions positively affecting growth and biomass accumulation did not promoted polymer yield increase [54,55,56,57]. The highest PHBV synthesis was observed in the range of 400–500 rpm (1.18 ± 0.06–1.27 ± 0.09 g/L). Stirring at values higher than 500 rpm did not cause further increases in PHBV production (Table 2).

### 3.4. PHBV Characterization

#### 3.4.1. DSC

**Figure 4:**
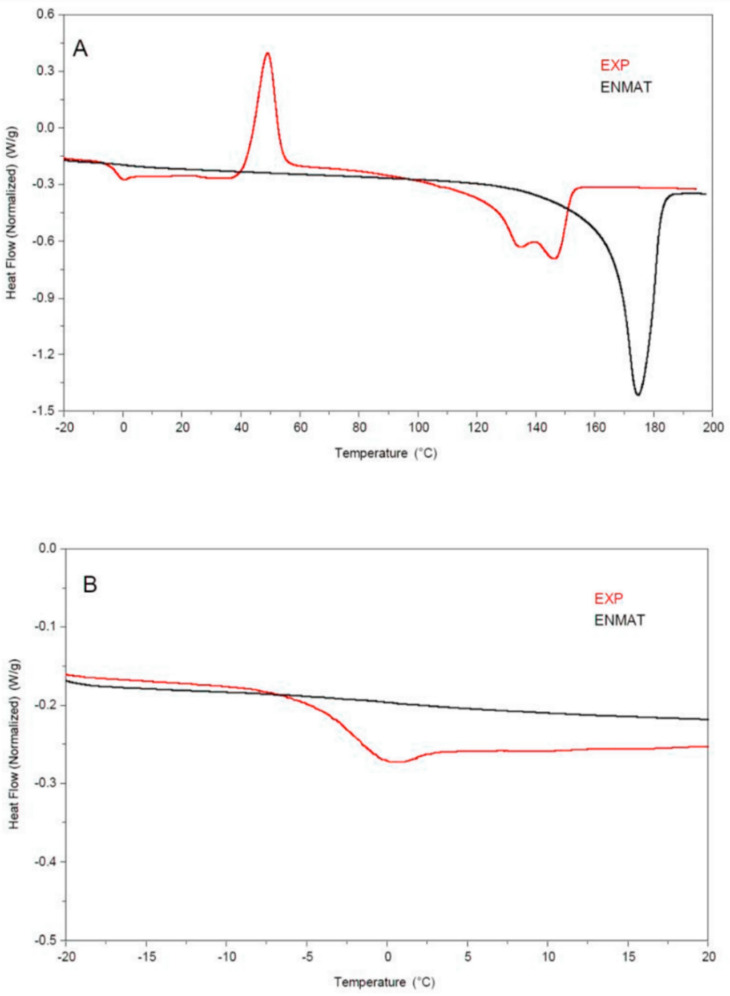
DSC thermograms (−20–200 °C, panel **A**; −20–20 °C, panel **B**) obtained from the second heating of the PHBV purified from R-NF fermented with *Haloferax mediterranei* DSM1411 (EXP) and the commercial reference ENMAT™ Y1000P (ENMAT).

PHBV produced under bioreactor system was collected and characterized. The thermodynamic parameters of the sample were compared to a commercial PHBV. Representative DSC curves and the thermal results obtained for the two polymer samples are reported in Figure 4A and Table 3, respectively.

**Figure 5:**
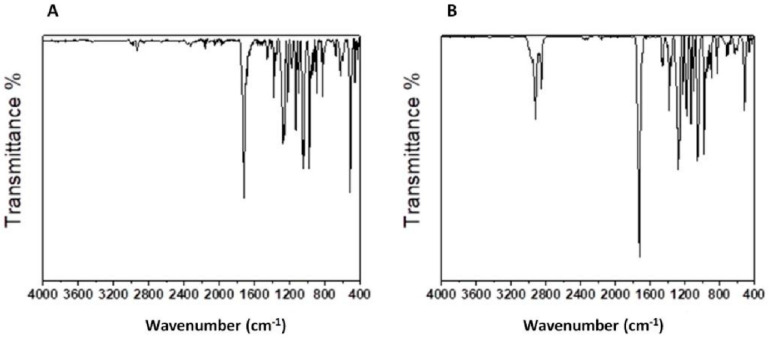
Attenuated total reflectance-Fourier transform infrared (ATR-FTIR) spectra of the PHBV purified from R-NF fermented with *Haloferax mediterranei* DSM1411 (panel **A**) and of the commercial reference ENMAT™ Y1000P (**B**).

The commercial ENMAT sample was characterized by a single endothermic melting peak (T_m_) at 175 °C, according to previously reported thermal data [58,59], while a double melting signal was observed for the polymer (EXP) synthesized by *Hfx. mediterranei* in R-NF. Moreover, the melting temperature of the experimental sample (T_m2_ = 146 °C) was significantly (*p* < 0.05) lower than that of the commercial PHBV. The glass transition temperature of PHBV samples was detected at low temperatures (Table 3 and Figure 5B).

In particular, the detection of the T_g_ of the reference PHBV was difficult due to the very weak intensity of the DSC signal, this dependent on the high crystallinity of material and the small heat capacity change recorded crossing the T_g_ [59,60]. Conversely, the T_g_ of the experimental PHBV was easily detected thanks to the relevant increase of the heat capacity across the glass transition, thus hypothesizing a lower crystallinity of the polymer compared to the reference.

#### 3.4.2. ATR-FTIR Spectroscopy

The purified polymer was analyzed by the FTIR spectrophotometer with ATR (Figure 5). The major absorption peaks of *Hfx. mediterranei* PHBV on FTIR spectrum are reported in Table 4.

FTIR spectra of both the samples showed an absorption peak near 1718 cm^−1^ which corresponds to the ester carbonyl bond (C=O), the most important feature of the PHBV [61,62]. Other relevant peaks for the polymer sample obtained under the conditions of this study (Figure 5 and Table 4) were found in the range 2913–2850, 1450–1380, at 1128 and in the range 973–821 cm^−1^ (the assigned type of vibrations is reported in Table 4).

The spectra corresponded to the typical profile of a copolymer 3-hydroxybutyrate (3HB) *co* 3-hydroxyvalerate (3HV). Indeed, similar observations were previously reported [62,63,64]. Compared to the commercial sample, a higher intensity of the branched alkyl fraction (2913–2850 cm^−1^) was observed (Figure 5), thus hypothesizing the higher presence of the poli-3-HV fraction in the copolymer.

#### 3.4.3. XPS Analysis

The XPS analysis investigated the surface characteristics of PHBV films. The calculated atomic percentages of each species of the sample are reported in Table 5.

All the elements besides C and O are polymer contaminants. In particular, the commercial ENMAT preparation was characterized by the presence of significantly (*p* < 0.05) higher percentages of N and Si. F, that was detected in both samples, derived from the release agent used for the obtaining the PHBV films.

**Figure 6:**
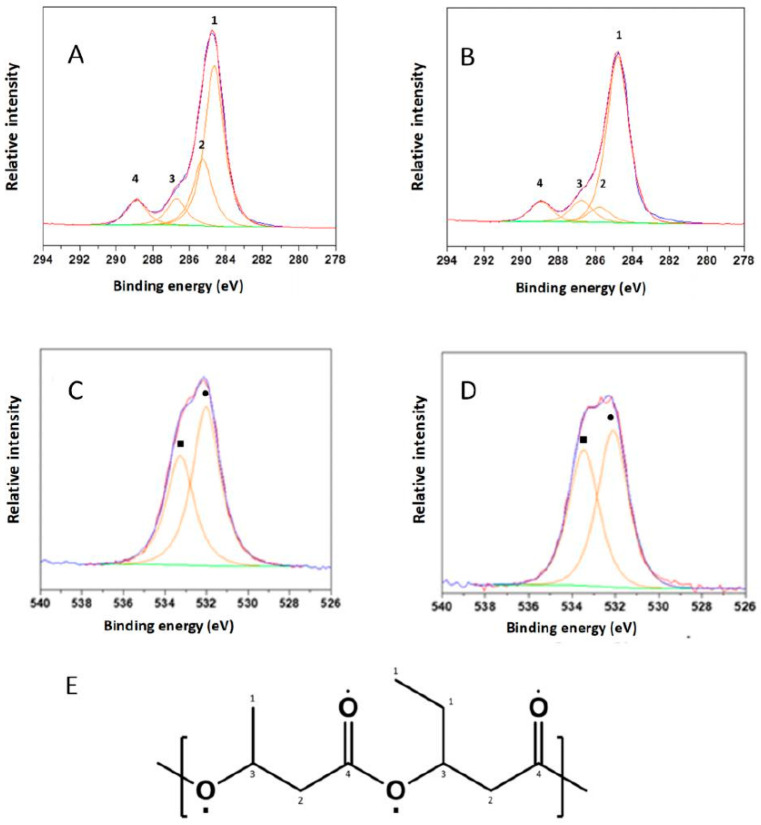
XPS spectral analysis on the surface of PHBV films. C1s spectra: (**A**), ENMAT and (**B**), PHBV from fermented R-NF; O1s spectra: (**C**), ENMAT and (**D**) from fermented R-NF. Peaks numbers and symbols correspond to functional groups reported in the structure of the PHBV polymer represented in panel (**E**). Relative abundances are detailed in Table 6.

The chemical environment of the atoms has been also investigated. The analysis of the spectra of the 1s orbital electrons of carbon (C 1s) (Figure 6A,B) showed a significantly (*p* < 0.05) higher abundance of the chemical environment related to C-H, C-C (peak at 284.8 ± 0.1 eV) in the PHBV purified from the fermented R-NF compared to the commercial preparation (Figure 6E and Table 6), thus confirming the consistent presence of the alkyl fraction.

Moreover, the O1s spectra (signals observed around binding energies of 532 eV, Figure 6C,D) of the PHBV purified from fermented R-NF revealed a higher (*p* < 0.05) abundance of the chemical environment related to the O-C functional group (533.4 ± 0.3 eV, relative abundance 46.4 ± 0.5 vs. 40.7 ± 0.5%) compared to the commercial PHBV sample (Figure 6E and Table 6).

#### 3.4.4. Mechanical Analysis

The mechanical properties of the purified PHBV produced under bioreactor system were investigated. The tensile strength resulted 22.7 ± 1.2 MPa, while the elongation break resulted 9.98 ± 0.32%. The Young’s modulus was 1.32 ± 0.22 GPa. Compared to the commercial preparation, tensile strength and Young’s modulus of the polymer produced by *H. mediterranei* were significantly (*p* < 0.05) lower, while the elongation break resulted significantly higher. In details, mechanical parameters for ENMAT were 29.5 ± 1.2 MPa (tensile strength), 3.64 ± 0.24% (elongation break), and 3.32 ± 0.22 GPa (Young’s modulus).

## 4. Discussion

Among halophiles, the haloarchaeon *Haloferax mediterranei* has been extensively studied for efficient PHA production. It has several advantages such as adaptivity, high growth rate, genetic stability, and an efficient synthesis of the polymer [65]. It was also demonstrated that *Hfx. mediterranei* can efficiently use carbon sources from different industrial and household wastes for synthesizing PHA.

Detailed investigations already revealed that the PHA produced by *Hfx. mediterranei* is PHBV, a copolymer of 3HB and 3HV from unrelated carbon sources [66]. Moreover, *Hfx. mediterranei* is also capable to synthesize poly(3-hydroxybutyrate-*co*-3-hydroxyvalerate-*co*-4-hydroxybutyrate) (PHBV4HB) [31,67].

PHBV is a polymer technologically and commercially preferred to PHB [28]. Most organisms require 3HV precursor for PHBV synthesis whereas *Hfx. mediterranei* can efficiently synthesize PHBV without any external precursor, thus greatly reducing the production cost [68].

It was reported that three important factors mainly contribute to the cost of PHA production: substrate usage, fermentation process, and PHA recovery [31]. Because PHA recovery from halophiles easily carried out by cell lysis using tap-water, the role of substrate and fermentation conditions in the process optimization is of great importance.

A possible strategy to overcome the challenge of an efficient PHA production is the usage of low-cost substrate which can compensate the high production cost resulting from low productivity. Indeed, it was estimated that the substrates account for almost 40–48% of the PHA production cost [69]. Agri-industrial wastes such as vinasse [70], olive mill wastewater [57], chitin waste from seafood industry [71] have been already tested as carbon substrate for PHA synthesized by *Hfx. mediterranei*.

RCEW, one of the by-products of the dairy industry, in an inexpensive and abundant substrate rich in nutrients. Although RCEW is often considered very similar to cheese whey, the concentration of its constituents is unavoidably lower, except for the ash content, which is affected by the acid and salt added to enhance the whey proteins flocculation and aggregation [72]. Moreover, the RCEW pH resulted higher than that of whey for the same reason (e.g., correction with sodium bicarbonate to improve the whey flocculation), while fat is quite completely retained during ricotta manufacturing [72].

According to previous findings [72], the multi-step fractionation described in this work allowed the retaining of the fat in the UF retentate and the separation of a lactose-rich from a protein-rich fractions. The former, containing 12.6% (*w*/*v*) of lactose, was used as substrate for PHA production; the latter can be easily subjected to the recovery of whey proteins to be used as food and feed supplements.

Moreover, the protein removal lead to the decrease of the nitrogen available during fermentation, hence avoiding the exo-polysaccharides (EPS) synthesis (which synthesis decrease the PHA production efficiency) and moving the microbial metabolism towards the bioplastic synthesis [73].

Since *Hfx. mediterranei* was unable to utilize lactose, enzymatically hydrolyzed R-NF was used as the sole carbon source. The enzymatic hydrolysis was preferred to acid one (that also requires high temperature) since more sustainable from both technical and economic point of views [74].The enzyme was chosen among commercial and relatively cheap preparations commonly used in dairy processes to hydrolyze lactose in glucose and galactose for lactose-free food production. The ability of *H. mediterranei* to metabolize both glucose and galactose was previously reported [75]. Micronutrients were additionally supplemented to favor sugars utilization for PHBV synthesis [76].

Optimization of the fermentation conditions (process parameters and substrate formula) is also the key to improve PHA production. Indeed, it is reported that the major drawbacks faced by haloarchaea species, such as *Hfx. mediterranei*, are the low PHA productivity resulted from slow growth rate and failing to achieve high cell-density cultivation [37].

The optimization of the fermentation conditions followed the classical approach of the one-factor-at-a-time (OFAT) experiments, which considered the variation of one factor at a time [77]. The lactose concentration of the R-NF fraction is the range previously reported as optimal for PHA synthesis through *Hfx. mediterranei* [26,67]; therefore, it was not changed through optimization process. Overall, *Hfx. mediterranei* undergoes temperature-driven fast metabolism which causes nitrogen deficiency and triggers PHA overproduction [37]. Nevertheless, a significant decrease in polymer production was found at 45 °C compared to 37 °C. Moreover, according to the early study of Fernandez-Castillo et al. [78], the PHA accumulation was higher when only 10% (*w*/*v*) NaCl was added to the RCEW fraction.

The addition of yeast extract, rich in free amino acids and minerals, did not improve PHA synthesis, since probably the supplementations altered the C/N ratio of the medium decreasing the polymer productivity by promoting EPS synthesis [73].

Once formula and temperature were set, the production of PHA in a bioreactor under stable and monitored conditions of pH, temperature, and stirring was tested. Overall, the amount of the polymer recovered at the end of fermentation was higher than that obtained in flasks, reaching amounts higher than 1 g/L of fermented R-NF. A similar experimental design for the optimization of the PHA synthesis through R-NF fermentation was carried out by the authors with a similar approach by using *Azotobacter vinelandii* UWD [79] as producer microorganism; nevertheless, the polymer production in any of the tested conditions, was lower than 127 ± 5 mg/L.

The chemical structure, surface condition of the polymer, and their related physical and thermal properties have also been investigated. Indeed, crystallinity, crystal structures, molecular orientation, melting temperature (T_m_), and glass transition temperature (T_g_) are known to have crucial effects on the PHAs biodegradation of polyesters [80]. In this study, the DSC analysis was used to study the thermal properties (T_g_, T_m_, and *X*) of the polymer synthesized by *Hfx. mediterranei* in R-NF, in comparison to a commercial PHBV sample. Overall, the polymer thermal behavior observed under this study conditions reflected data previously obtained for PHBV [50], confirming its identity. Nevertheless, the experimental sample was characterized by a double endothermic melting peak, which can be attributed to the presence of two crystalline phases of different sizes, orders, and thickness [81,82].

Previous studies demonstrated that increasing ratio of HV fraction in the PHBV causes the decrease of the melting temperature [83]. The T_m_ of the PHBV here synthesized by *Hfx. mediterranei* was markedly lower than the commercial sample, this latter previously characterized by the 1–3 mol% HV [84,85]. According to the literature [86], melting point of the experimental PHBV corresponds to an approximately HV content of 16 mol%. Moreover, thermal behavior showed that experimental PHBV was characterized by a low crystallinity, a characteristic associated to improved degradation rate and the processability of the PHBV polymer [80,87].

The results of the X-ray photoelectron spectroscopy analysis, providing more quantitative measurement than FTIR, was used to investigate elemental composition of the PHBV synthesized by *Hfx. mediterranei* in R-NF. Compared to the commercial sample, a lower contamination of the polymer synthesized in R-NF was observed. Such contaminations are commonly found in different commercial PHA samples as residues of the extraction and purification protocols and reagents [88].

Overall, the XPS results corroborated the DSC and ATR-IFTR conclusions regarding the abundance of the alkyl region, corresponding to the HV chains. The analysis of the mechanical properties revealed a lower tensile strength than the commercial sample, this latter characterized by a low percentage of HV (1–3 mol%) [84,85]. It was previously reported that increasing HV percentages in the PHBV co-polymer cause the decrease of the tensile strength. Nevertheless, a relevant increase of the elongation break was observed in correspondence of high HV incorporation [85]. Indeed, a, three-fold higher elongation break value was observed for the experimental PHBV sample compared to ENMAT commercial preparation.

## 5. Conclusions

PHA has largely been recognized as an emerging group of biopolymers suitable for multiple applications instead of the conventional petroleum-derived plastics. Among the different microorganisms recently investigated for PHA biosynthesis, *Hfx. mediterranei,* able to accumulate PHBV, is considered the most promising for the large-scale production. Aiming at solving the economical uncompetitiveness of the bioplastic production, RCEW, considered as one of the large wastewater from the agri-food compart, was proposed as a sustainable carbon source for the synthesis of the polymer. A membrane multi-step fractionation was used for lactose enrichment (and contemporary recover whey proteins), and process conditions were optimized to maximize the *Hfx. mediterranei* synthesis of PHBV, confirming the validity of the biotechnological process. Further investigation will clarify the peculiarities in terms of thermal/mechanical properties of the polymer synthesized under the study conditions by *Hfx. mediterranei*, thus hypothesizing proper industrial uses. At the same time, the evaluation of fed-batch or continuous fermentation conditions could increase the potential of the bioprocess proposed, allowing the developing a large-scale bioplastic production.

## Figures and Tables

**Table 1:** Proximal composition of RCEW, and R-UF, P-UF, and R-NF fractions. Protein content is calculated as Total Nitrogen *×* 6.38.

	RCEW	R-UF	P-UF	R-NF
pH	5.2 ± 0.2 ^a,b^	5.2 ± 0.1 ^b^	5.2 ± 0.1 ^b^	5.6 ± 0.2 ^a^
Total Nitrogen (mg/L)	59.12 ± 1.23 ^b^	285.12 ± 8.09 ^a^	1.36 ± 0.08 ^d^	5.07 ± 0.15 ^c^
Proteins (%*w*/*v*)	0.38 ± 0.06 ^b^	1.81 ± 0.05 ^a^	0.08 ± 0.01 ^c^	0.03 ± 0.01 ^c^
Total Free Amino Acids (mg/L)	420 ± 12 ^c^	50 ± 5 ^d^	510 ± 15 ^b^	1897 ± 21 ^a^
Lactose (%*w*/*v*)	3.78 ± 0.05 ^c^	5.2 ± 0.1 ^b^	3.41 ± 0.07 ^d^	12.6 ± 0.07 ^a^
Glucose (%*w*/*v*)	<0.01	<0.01	<0.01	<0.01
Galactose (%*w*/*v*)	<0.01	<0.01	<0.01	<0.01
Fat (%*w*/*v*)	0.16 ± 0.10 ^b^	0.75 ± 0.04 ^a^	<0.01	<0.01
Ash (%*w*/*v*)	1.10 ± 0.10 ^b^	0.22 ± 0.05 ^c^	1.32 ± 0.09 ^a^	0.32 ± 0.13 ^c^

The data are the means of three independent experiments ± standard deviations (*n* = 3). ^a–c^ Values in the same raw, with different superscript letters, differ significantly (*p* < 0.05).

**Table 2:** CDM, PHBV production and yield obtained after 72 h of fermentation in bioreactor system.

Stirring	CDM (g/L)	PHBV (g/L)	Yield (% *w*/*w*)
300 rpm	10.75 ± 0.14 ^d^	0.94 ± 0.05 ^b^	8.74 ± 0.09 ^a^
400 rpm	12.66 ± 0.17 ^c^	1.18 ± 0.06 ^a^	9.36 ± 0.55 ^a^
500 rpm	18.32 ± 0.15 ^a^	1.27 ± 0.09 ^a^	7.03 ± 0.32 ^b^
600 rpm	14.65 ± 0.10 ^b^	0.95 ± 0.05 ^b^	6.48 ± 0.12 ^c^

The data are the means of three independent experiments ± standard deviations (*n* = 3). ^a–d^ Values in the same column, with different superscript letters, differ significantly (*p* < 0.05).

**Table 3:** Thermal properties of the PHBV purified from R-NF fermented with *Hfx. mediterranei* DSM1411 (EXP) and the commercial reference ENMAT™ Y1000P, as obtained by the differential scanning calorimetry (DSC) analysis. T_g_, glass transition temperature; T_c_, crystallization temperature; T_m_, melting temperature; ΔH_m_ and ΔH_m_, melting and crystallization enthalpies; *X* crystallinity degree.

	T_g_ (°C)	T_c_ (°C)	T_m1_ (°C)	T_m2_ (°C)	H_c_ (J/g)	H_m_ (J/g)	*X* (%)
EXP	−3.0 ± 0.2 ^b^	49.2 ± 0.4	135.2 ± 0.5	146.2 ± 0.4 ^b^	34.2 ± 0.2	62.3 ± 0.3 ^b^	26.2 ± 0.2 ^b^
ENMAT	1.1 ± 0.2 ^a^	-	-	175.3 ± 0.3 ^a^	-	96.1 ± 0.4 ^a^	88.5 ± 0.3 ^a^

The data are the means of three independent experiments ± standard deviations (*n* = 3). ^a,b^ Values in the same column, with different superscript letters, differ significantly (*p* < 0.05).

**Table 4:** Absorption peaks of the PHBV purified from R-NF fermented with *Hfx. mediterranei* DSM1411.

Spectral Region (cm^−1^)	Type of Vibration in the Functional Group
2913–2850	(CH, CH2) symmetric and asymmetric stretching
1718	(C=O) stretching
1450–1380	(C-C) stretching
1128	(C-O) stretching
973–821	(C-C) deformation

**Table 5:** Atomic percentages, based on the spectral analysis, of the surface of PHBV film purified from R-NF fermented with *Hfx. mediterranei* DSM1411 (EXP) and of the commercial reference ENMAT™ Y1000P.

	Atomic Percentage	
Element	ENMAT	EXP
C	80.7 ± 0.2 ^b^	83.4 ± 0.2 ^a^
O	16.4 ± 0.2 ^a^	15.1 ± 0.2 ^b^
N	1.4 ± 0.2 ^a^	0.3 ± 0.2 ^b^
F	0.4 ± 0.2 ^a^	0.5 ± 0.2 ^a^
Si	1.1 ± 0.2 ^a^	<0.2 ^b^
Na	<0.2	<0.2
S	<0.2	nd
Ca	nd	<0.2
P	nd	<0.2
B	nd	nd

The data are the means of three independent experiments ± standard deviations (*n* = 3). ^a,b^ Values in the same raw, with different superscript letters, differ significantly (*p* < 0.05). nd: not detected.

**Table 6:** Spectral components and relative abundance (%) of the surface of PHBV film purified from R-NF fermented with *Hfx. mediterranei* DSM1411 (EXP) and of the commercial reference ENMAT™ Y1000P, as obtained by XPS analysis.

Peak *	Position (eV)	Relative Abundance (%)	
		ENMAT	EXP
**C 1s Spectra**			
1	284.8 ± 0.1	57.6 ± 0.5 ^b^	74.6 ± 0.5 ^a^
2	285.6 ± 0.3	23.9 ± 0.5 ^a^	6.5 ± 0.5 ^b^
3	286.9 ± 0.2	9.5 ± 0.5 ^a^	9.8 ± 0.5 ^a^
4	289.0 ± 0.2	9.0 ± 0.5 ^a^	9.1 ± 0.5 ^a^
**O 1s Spectra**			
•	531.8 ± 0.3	59.3 ± 0.5 ^a^	53.7 ± 0.5 ^b^
■	533.4± 0.3	40.7 ± 0.5 ^b^	46.4 ± 0.5 ^a^

* Peak symbols refer to Figure 6E. The data are the means of three independent experiments ± standard deviations (*n* = 3). ^a,b^ Values in the same raw, with different superscript letters, differ significantly (*p* < 0.05).

## Supplementary Materials

The following are available online at https://www.mdpi.com/2304-8158/9/10/1459/s1, Figure S1: Schematic representation of the bioreactor. Figure S2: Residual lactose (% *w*/*v*) in R-NF fraction after treatments with commercial β-galactosidase preparations. LGI100, 400, 1000: Maxilact LGI 5000 added at 100, 400, or 1000 µL/100 mL; A4100, 400, 1000: Maxilact A4 added at 100, 400, or 1000 µL/100 mL. Not treated R-NF fraction was used as control (100%). Error bars represent the standard deviation of three replicates. a–f Values with different superscript letters, differ significantly (*p* < 0.05). Table S1: Formulations for Halobacterium medium 97 and 372.

## Abbreviations

RCEW	ricotta cheese exhausted whey
COD	chemical oxygen demand
BOD	biochemical oxygen demand
PHA	poly-hydroxyalkanoates
UF	ultra-filtration
NF	nano-filtration
TN	total nitrogen
VCR	Volume Concentration Ratio
R-UF	ultra-filtration retentate
P-UF	ultra-filtration permeate
FAA	free amino acids
TFAA	total free amino acids
R-NF	nano-filtration retentate
P-NF	nano-filtration permeate
PHBV	poly(3-hydroxybutyrate-co-3-hydroxyvalerate)
CDM	cell dry mass
HM97	Halobacterium Medium 97
HM372	Halobacterium Medium 372
YE	yeast extract
DSC	Differential scanning calorimetry
ATR-FTIR	Attenuated Total Reflectance-Fourier Transform Infrared
3HB	3-hydroxyvalerate
XPS	X-ray Photoelectron Spectroscopy
PHB	polyhydroxybutyrate
PHBV4HB	poly(3-hydroxybutyrate-co-3-hydroxyvalerate-co-4-hydroxybutyrate)
EPS	exo-polysaccharides
OFAT	one-factor-at-a-time
TMP	Trans Membrane Pressure
