# Supplementary material for: Production of the Polyhydroxyalkanoate PHBV from Ricotta Cheese Exhausted Whey by Haloferax mediterranei Fermentation
Source: Foods. 2020 Oct 14;9(10):1459. doi: 10.3390/foods9101459 (PMC7602231; doi:10.3390/foods9101459)

Table S1. Formulations for Halobacterium medium 97 and 372

	**Halobacterium medium 97** (g)	**Halobacterium medium 372** (g)
Casamino acids	7,5	5
Yeast extract	10	5
Na_3-citrate	3	3
KCl	2	2
$MgSO_4$ x 7 H_2O	20	20
$FeSO_4$ x 7 H_2O	0,05	------
FeCl2 x 4 H_2O	------	$36 \cdot 10^{-3}$
$MnSO_4$ x H_2O	$0,2 \cdot 10^{-3}$	------
MnCl2 x 4 H_2O	------	$0,36 \cdot 10^{-3}$
NaCl	250	200
Na-glutamate	------	1
Distilled water	1000 mL	1000 mL

Figure S1. Residual lactose (%) in R-NF fraction after treatments with commercial β-galactosidase preparations. $LGI_{100,\ 400,\ 1000}$: Maxilact LGI 5000 added at 100, 400, or 1000 μL/100mL; $A4_{100,\ 400,\ 1000}$**:** Maxilact A4 added at 100, 400, or 1000 μL/100mL. Not treated R-NF fraction was used as control (100%). Error bars represent the standard deviation of three replicates. [a-f]Values with different superscript letters, differ significantly (P<0.05).

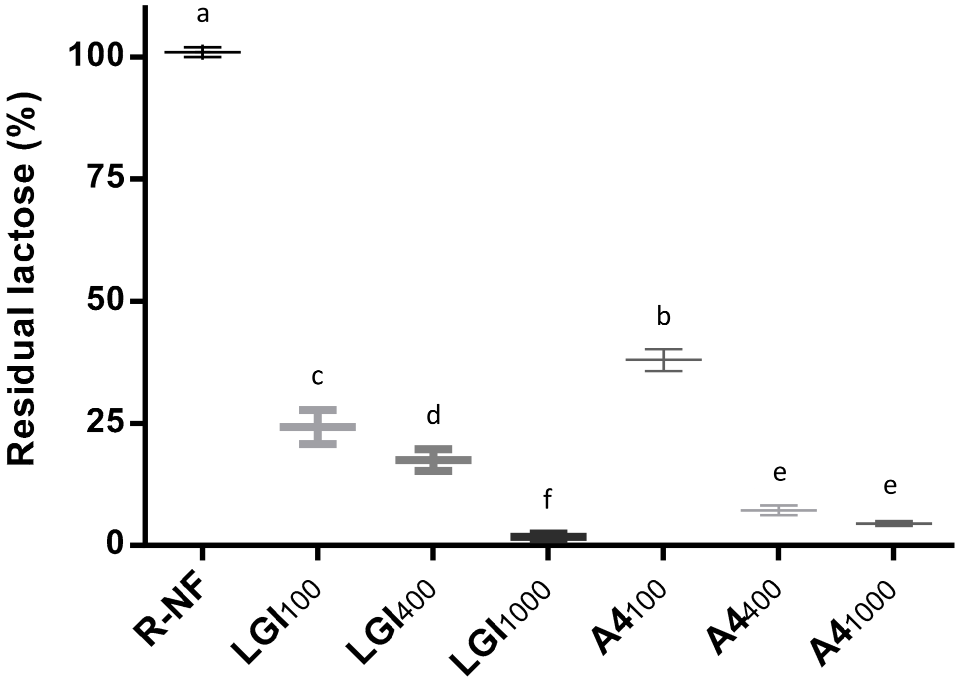

Supplement: Supplementary file 1 [file foods-09-01459-s001.pdf]
